# Post-weaning A1/A2 β-casein milk intake modulates depressive-like behavior, brain μ-opioid receptors, and the metabolome of rats

**DOI:** 10.1016/j.isci.2021.103048

**Published:** 2021-08-28

**Authors:** Aya Osman, Simone Zuffa, Gemma Walton, Elizabeth Fagbodun, Panos Zanos, Polymnia Georgiou, Ian Kitchen, Jonathan Swann, Alexis Bailey

**Affiliations:** 1 Department of Psychiatry, Icahn School of Medicine at Mount Sinai, New York, NY 10029, USA; 2 Seaver Autism Center for Research and Treatment, Icahn School of Medicine at Mount Sinai, New York, NY 10029, USA; 3 Department of Metabolism, Digestion and Reproduction, Faculty of Medicine, Imperial College London, London, UK; 4 Food and Nutritional Sciences, School of Chemistry, Food and Pharmacy, University of Reading, Reading, UK; 5 Pharmacology Section, Institute of Medical and Biomedical Education, St George’s University of London, London, UK; 6 Department of Psychology, University of Cyprus, 1 University Avenue, 2109 Nicosia, Cyprus; 7 Department of Psychiatry, School of Medicine, University of Maryland, Baltimore, MD, USA; 8 School of Biosciences and Medicine, Faculty of Health and Medical Sciences, University of Surrey, Guildford, Surrey GU2 7XH, UK; 9 School of Human Development and Health, Faculty of Medicine, University of Southampton, Southampton, UK

**Keywords:** Nutrition, Neuroscience, Microbiome

## Abstract

The postnatal period is critical for brain and behavioral development and is sensitive to environmental stimuli, such as nutrition. Prevention of weaning from maternal milk was previously shown to cause depressive-like behavior in rats. Additionally, loss of dietary casein was found to act as a developmental trigger for a population of brain opioid receptors. Here, we explore the effect of exposure to milk containing A1 and A2 β-casein beyond weaning. A1 but not A2 β-casein milk significantly increased stress-induced immobility in rats, concomitant with an increased abundance of *Clostridium histolyticum* bacterial group in the caecum and colon of A1 β-casein fed animals, brain region-specific alterations of μ-opioid and oxytocin receptors, and modifications in urinary biochemical profiles. Moreover, urinary gut microbial metabolites strongly correlated with altered brain metabolites. These findings suggest that consumption of milk containing A1 β-casein beyond weaning age may affect mood via a possible gut-brain axis mechanism.

## Highlights

• Postnatal brain development is sensitive to nutritional exposures
• Consumption of A1 but not A2 β-casein milk post-weaning affects mood in rats
• Gut microbial, biochemical, and neurochemical changes accompany mood alterations
• Urinary gut microbial metabolites correlate with brain metabolites

## Graphical abstract

**Figure:**
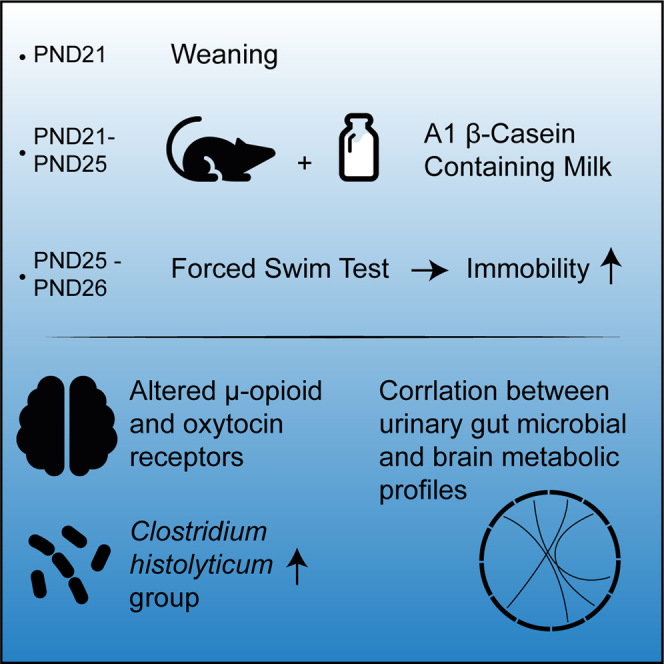

## Introduction

During the first 2 years of life, the human brain undergoes a period of rapid growth and development, which is sensitive to both genetic and environmental stimuli (Chen and Baram, 2016). Nutrition has an influential role in this process with evidence suggesting variations in dietary exposures during this developmental window can contribute to changes in brain structure and function with consequences for mental health disorders (Bolton and Bilbo, 2014; Borre et al., 2014). Milk is the primary source of nutrients in mammals during early life and the removal of milk and introduction of a solid diet, i.e. the weaning process, represents a major nutritional event during infancy. While weaning practices vary among the human population, with differences in age of weaning and type of milk consumed, the influence of such variables on brain development, structure, function, and behavior remains poorly understood.

Mammalian milk components have been shown to interact with and modify opioidergic systems in neonates (Brantl et al., 1979; Kitchen et al., 1995; Teschemacher et al., 1997). We have previously demonstrated in rats that weaning on postnatal day (PND) 21, the standard age of weaning in rodents (Cramer et al., 1990), acts as a signal for the developmental activation of a population of opioid receptor subtype (Kitchen et al., 1995). This effect was shown to be independent of psychological (maternal deprivation) or physiological (suckling) stimuli and is solely dependent on the loss of dietary casein (Goody and Kitchen, 2001). As the opioid system is known to play a crucial role in the regulation of emotional behavior (Lutz and Kieffer, 2013; Puryear et al., 2020), it was hypothesized that the weaning process may impact on such behavior. To this end, we have also demonstrated that preventing the weaning process for 5 days in rats resulted in increased stress-induced immobility during the forced swim test (FST), concomitant with a resistance to stress-induced modulation of oxytocin receptors (OTrs) in amygdala nuclei, indicative of passive stress-coping mechanisms (Farshim et al., 2016).

Emerging evidence suggests that the resident bacteria of the gastrointestinal tract (GI), collectively known as the gut microbiota, play a key role in the development of host’s brain and behavior through a mechanism known as the gut-brain axis (Cryan et al., 2019). These gut bacteria and their resultant metabolome sit at the interface of environmental dietary exposure and brain development. We therefore investigated the impact of weaning on gut bacterial, host, and integrated pan-kingdom metabolism. Indeed, restricting the weaning process was also found to alter the compositional structure of the intestinal microbiota and its metabolic exchange with the host (Farshim et al., 2016). Although the mechanism underlying these effects remains unclear, these findings suggest weaning may affect the maturation of the central nervous system by modulating the developing gut-brain axis.

Casein is one of the most abundant proteins found in milk, and it has been extensively studied for its ability to release peptide fragments with opioid activity upon proteolytic cleavage (Kamiński et al., 2007). The most widely studied group of these peptides is the β-casomorphins, which derive from milk β-casein and have been identified in bovine, ovine, and human milk (Teschemacher et al., 1997). β-Casein makes up 30% of the total protein content in bovine milk, and it is mainly present in two major genetic variants: A1 and A2 (Kamiński et al., 2007). These two variants differ in the presence of either histidine (His^67^) in A1 β-casein or proline (Pro^67^) in A2 β-casein at position 67 of this protein. The emergence of the A1 variant was consequential to a point mutation in ancestors to modern European-type cattle. As a consequence, commercially available milk in many Western countries contains a mixture of A1 and A2 β-casein (Pal et al., 2015). The proteolytic cleavage of A1 but not A2 β-casein during digestion by host and gut microbial enzymes results in the release of β-casomorphin-7 (BCM-7), a bioactive peptide (Jinsmaa and Yoshikawa, 1999) with a high affinity for μ-opioid receptors (MOPrs), which are not only expressed in the brain but also along the GI tract and various immune cells (Brantl et al., 1981; Teschemacher, 2005; Nam et al., 2019). Moreover, BCM-7 has been implicated in the pathophysiology of a wide range of disorders, including abnormal gastrointestinal function (Zoghbi et al., 2006), inflammation (Yadav et al., 2020), and mental health disorders such as schizophrenia and autism spectrum disorders (Reichelt et al., 1981; Jarmołowska et al., 2019).

Given the evidence of a potential role for milk casein to impact brain development and behavior, here we investigate the exposure to casein-rich milk containing both A1 and A2 β-casein variants and casein-free milk beyond the standard weaning age in rats and its influence on behavior, neurochemical, microbial, and metabolic profiles. To further elucidate the influence of these casein variants, the effect of milk consumption containing both A1 and A2 β-casein or exclusively A2 β-casein beyond weaning was also investigated.

## Results

### Consumption of milk containing casein beyond weaning age results in increased stress-induced immobility during the FST

**Figure 1:**
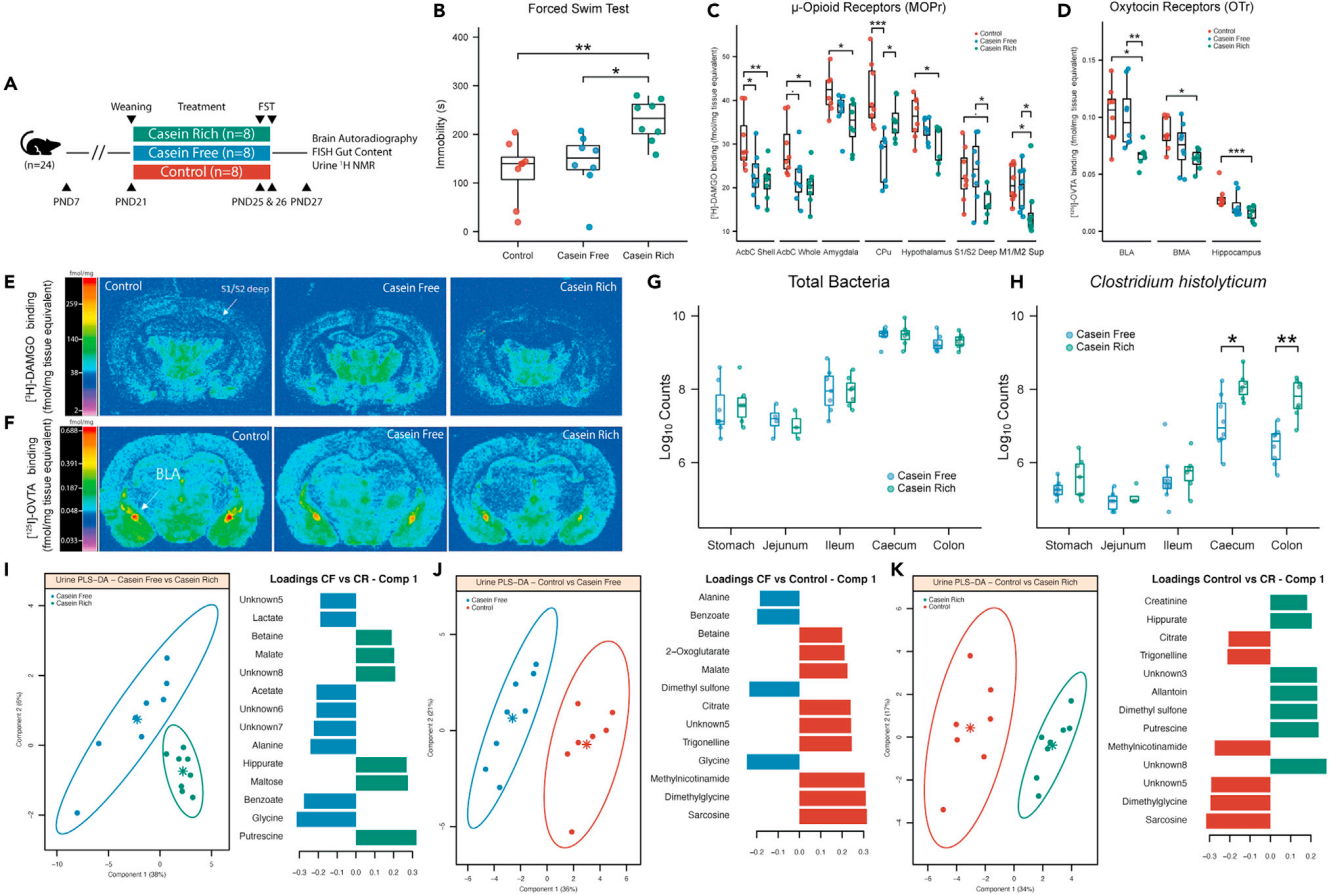
Milk casein consumption beyond weaning age results in increased stress-induced immobility, indicative of depressive-like behavior, alterations in brain μ-opioid (MOPr) and oxytocin receptors (OTr), gut microbial composition, and urine biochemical profiles (A) Study design. Rats were received at the experimental animal facility on PND7 and weaned on PND21. Divided in three groups, two received either casein-free or casein-rich milk while the control group received only water from PND21 to PND26. The FST was conducted on PND25 (pre-test) and PND26 (test). Urines were collected for 24 hr on PND27, before sacrifice, for ^1^H NMR spectroscopy analysis. The brain and gut were dissected to collect samples for receptor autoradiography and FISH analysis, respectively. (B) FST revealed significantly increased immobility time in the casein-rich group, indicative of depressive-like behavior, compared to both control (p = 0.004) and casein-free group (p = 0.016) (ANOVA followed by Tukey’s HSD post-hoc test). (C and D**)** μ-opioid (MOPr) and oxytocin receptors (OTr) autoradiography of different brain regions. Animals from the casein-rich group showed decreased MOPr with respect to control in the somatosensory cortex (S1/S2 Deep), hypothalamus, amygdala, nucleus accumbens (AcbC) whole and shell. Additionally, these animals presented significantly less OTr compared to control in BLA, basal medial amygdala (BMA), hippocampus, thalamus, and central nucleus of amygdala (CE) (ANOVA followed by Tukey’s HSD post-hoc test). (E and F**)** Computer-enhanced representative autoradiograms of adjacent coronal brain sections at the level of thalamus (bregma −2.80 mm). μ-opioid receptors (MOPrs) labeled with [^3^H]-DAMGO (4nM) showed decreased receptor binding in the deep layer of the somatosensory cortex of the casein-rich animals compared to control and casein-free. Oxytocin receptors (OTrs) labeled with [^125^I]-OVTA (50 pm) showed decreased receptor binding in the BLA casein-rich animals compared to control and casein-free. The color bar illustrates a pseudo-color interpretation of black and white film images in fmol/mg tissue equivalent. (G and H**)** FISH analysis revealed no differences in total bacteria abundance in any of the analyzed gut regions between casein-rich and casein-free animals. Nevertheless, the casein-rich group presented higher abundance of the *Clostridium histolyticum* group after FST in the caecum and colon compared to the casein-free group (Wilcoxon test followed by Benjamini-Hochberg correction). (I**–**K) Pairwise PLS-DA of urine biochemical profiles and loadings of metabolites with VIP>1. Urine of control animals were characterized by a higher presence of sarcosine, dimethylglycine, methylnicotinamide, trigonelline, and citrate compared to both the other groups. Rats fed with casein-rich milk excreted more putrescine and hippurate compared to the other groups while rats fed with casein-free milk excreted more glycine, benzoate, and alanine. Boxplots show first (lower) quartile, median, and third (upper) quartile. Significant and close to significant p values are represented as ⋅p < 0.1, ∗p < 0.05, ∗∗p < 0.01, ∗∗∗p < 0.001. Abbreviations: S1/S2, somatosensory cortex; AcbC, nucleus accumbens; Amy, amygdala; CPu, caudate putamen; BLA, basal lateral amygdala; BMA, basal medial amygdala; CE, central nucleus of amygdala.

To assess the role of milk casein in driving the phenotypic, biochemical, and microbial variation associated with delayed weaning, an animal study was performed with three groups of eight male, Wistar albino rats. These animals had free access to water, rat chow diet, and were housed with their cross-fostered mothers until PND21, at which point mothers were removed from the cage, effectively weaning the pups (Figure 1A). The control group was maintained on standard drinking water from PND21 to PND25. The casein-rich group was provided with a milk formula containing ∼20% (w/w) fresh weight casein (4.24% w/v casein when in liquid form), reflective of slightly higher casein levels than normally found in bovine milk (Roy et al., 2020) and thus defined as casein-rich milk (see Table S2 for milk nutritional information). The third group was provided with milk lacking casein, with total protein levels maintained using soya as a replacement protein, hence named casein-free milk (see Table S2 for milk nutritional information). Of note, casein was exclusively present in the milk source. The casein-rich group was observed to collectively consume a greater amount of milk compared to casein-free counterparts (Figure S1A) but did not gain significantly more weight compared to other groups (Figure S1B). During the FST, the total amount of time the rat spent still, not attempting to escape from the water, was measured and reported as total immobility time. Under normal physiological conditions, animals display escape-like behaviors during the FST, and antidepressants are known to increase this measure in rats (Detke et al., 1995; Lino-De-Oliveira et al., 2005). Animals fed casein-rich milk displayed significantly increased immobility time compared to both control (p < 0.01; analysis of variance [ANOVA] followed by Tukey’s HSD post-hoc test) and casein-free groups (p < 0.05), indicative of a depressive-like phenotype (Figure 1B).

To confirm that the observed depressive-like phenotype derived from the consumption of higher levels of milk casein, we conducted a dose response study with different concentrations of milk casein, using an identical protocol to the aforementioned study. Specifically, identical casein-free formula utilized in the previous study (Special Diet Services, Cambridge, UK) was spiked with 10% or 20% w/w casein (Sigma, Poole, UK) in milk powder. A control group provided with un-spiked casein-free milk was also included. During the FST, a clear concentration-dependent relationship between milk casein and immobility behavior was observed, with the 20% w/w group but not the 10% w/w group displaying an increased immobility time compared to control (Figure S2A). This is consistent with the casein-rich vs casein-free observations. Both 10% and 20% w/w groups gained significantly more weight and consumed more milk overall compared to the control group (Figures S2B and S2C), also consistent with the observed trends in the previous study.

### Casein consumption modulates opioid and oxytocin receptor systems in specific brain regions

To assess if milk casein exposure beyond weaning age alters brain neurochemistry, potentially contributing to the behavioral changes observed in the FST, quantitative autoradiographic binding of MOPrs and OTrs was performed. These receptors were selected as they are sensitive to alterations induced by weaning and due to their involvement in emotional regulation (Lutz and Kieffer, 2013; Zanos et al., 2014). MOPr binding was carried out using [^3^H]-DAMGO on coronal sections cut across the whole brain. Reduced MOPr binding in the deep layer of somatosensory cortex was observed in casein-rich group compared to the casein-free counterpart (p < 0.05; ANOVA followed by Tukey’s HSD post-hoc test) (Figures 1C and 1E). Casein was also found to decrease MOPr binding in the hypothalamus (p < 0.05) and amygdala (p < 0.05) compared to control animals. Both casein-rich and casein-free milk caused a decrease in MOPr binding in the whole nucleus accumbens (p < 0.01 and p < 0.05, respectively) and its shell (p < 0.01 and p < 0.05 respectively), indicating a general milk effect, independent of casein, on MOPr binding in these regions. Meanwhile, casein-free milk was found to reduce MOPr binding in the caudate putamen (CPu) compared to both control (p < 0.001) and casein-rich group (p < 0.05) (Figure 1C). The effect of casein-rich or casein-free treatment was not found to be significant in the rest of the regions analyzed (Figure S3A). OTr autoradiographic binding was carried out using [^125^]-OVTA on coronal rat brain sections. Reduced OTr binding in the basal lateral amygdala (BLA) was observed in casein-rich treated animals compared to both control and casein-free counterparts (p < 0.05) (Figures 1D and 1F). Casein-rich milk also caused a significant reduction in OTr binding in the basal medial amygdala (BMA) (p < 0.05) and hippocampus (p < 0.001) compared to control (Figure 1C). The effects of casein-rich or casein-free treatments were not found to be significant in any of the other regions analyzed (Figure S3B).

### *C. histolyticum* group abundance in the caecum and colon increases following casein exposure and FST

Luminal contents from five different regions of the GI of the casein-rich and casein-free animals were collected on PND27, after FST, and analyzed with fluorescence *in situ* hybridization (FISH) to quantitatively assess the presence of specific gut bacteria in the samples. Casein intake was not found to alter the total count of bacteria in any of the analyzed regions (Figure 1G). Nevertheless, bacteria in the *Clostridium* cluster I and II (*Clostridium histolyticum* group) were significantly increased in the caecum and colon (p < 0.05 and p < 0.01, respectively; Wilcoxon test followed by Benjamini-Hochberg correction) of animals fed casein-rich milk compared to the casein-free group (Figure 1F). No significant differences were observed in the abundance of other early life-related gut bacteria, such as *Bifidobacterium* spp. and *Lactobacillus/Enterococcus* spp., between the two treatments in any of the examined regions (Figure S4).

### Casein-rich and casein-free milk modulate urinary metabolic profiles

The metabolic profiles of urine collected from control, casein-rich, and casein-free treated animals on PND27 were analyzed using ^1^H NMR spectroscopy. Pairwise supervised analysis using partial least squares-discriminant analysis (PLS-DA) confirmed statistical differences between the casein-rich, casein-free, and control groups. Compared to control, casein-rich milk intake resulted in an increase in the urinary excretion of hippurate, creatinine, allantoin, dimethyl sulfone, and putrescine and a decrease in the excretion of *N*-methylnicotinamide (NMND), trigonelline, dimethylglycine (DMG), citrate, and sarcosine (PLS-DA Q^2^Y = 0.77) (Figure 1I). Casein-free milk consumption resulted in a higher excretion of benzoate, glycine, dimethyl sulfone, and alanine and a reduced excretion of NMND, trigonelline, betaine, DMG, sarcosine, 2-oxoglutarate, malate, and citrate compared to control (PLS-DA Q^2^Y = 0.85) (Figure 1J). Finally, metabolites driving the separation between casein-rich and casein-free animals were hippurate, putrescine, maltose, malate, and betaine, both higher in animals fed casein-rich milk, and benzoate, glycine, acetate, lactate and alanine, which were in higher concertation in animals fed casein-free milk (PLS-DA Q^2^Y = 0.41) (Figure 1K).

**Figure 2:**
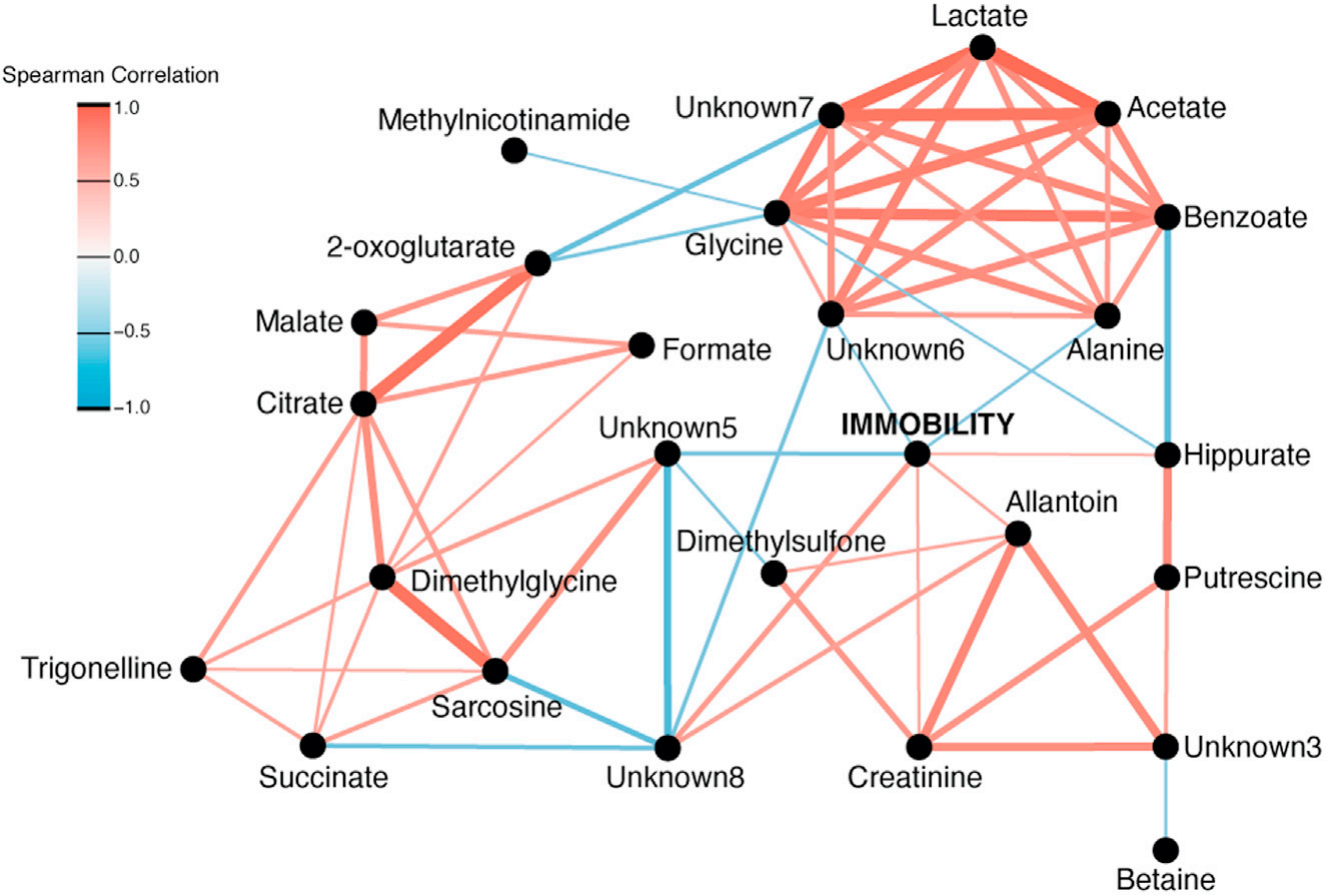
Correlation network between immobility and urine metabolites Immobility was positively correlated to creatinine, allantoin, and hippurate and negatively correlated to alanine. Significant Spearman correlations after Benjamini-Hochberg correction were plotted (p < 0.05). Red indicates positive correlation and blue negative correlation. Thicknesses of the edges are weighted on correlation intensity.

Specific urinary metabolites were associated with increased immobility time. A metabolic network was constructed using the Spearman correlation between immobility time and the metabolites. Immobility positively correlated with some microbial and endogenous metabolites such as hippurate (ρ = 0.55), allantoin (ρ = 0.56), and creatinine (ρ = 0.56) while it negatively correlated with alanine (ρ = −0.56). Sarcosine and acetate were negatively associated with immobility time via a number of known and unknown metabolites (Figure 2).

### Consumption of commercialized milk (A1/A2 β-casein) beyond weaning results in increased stress-induced immobility in the FST

**Figure 3:**
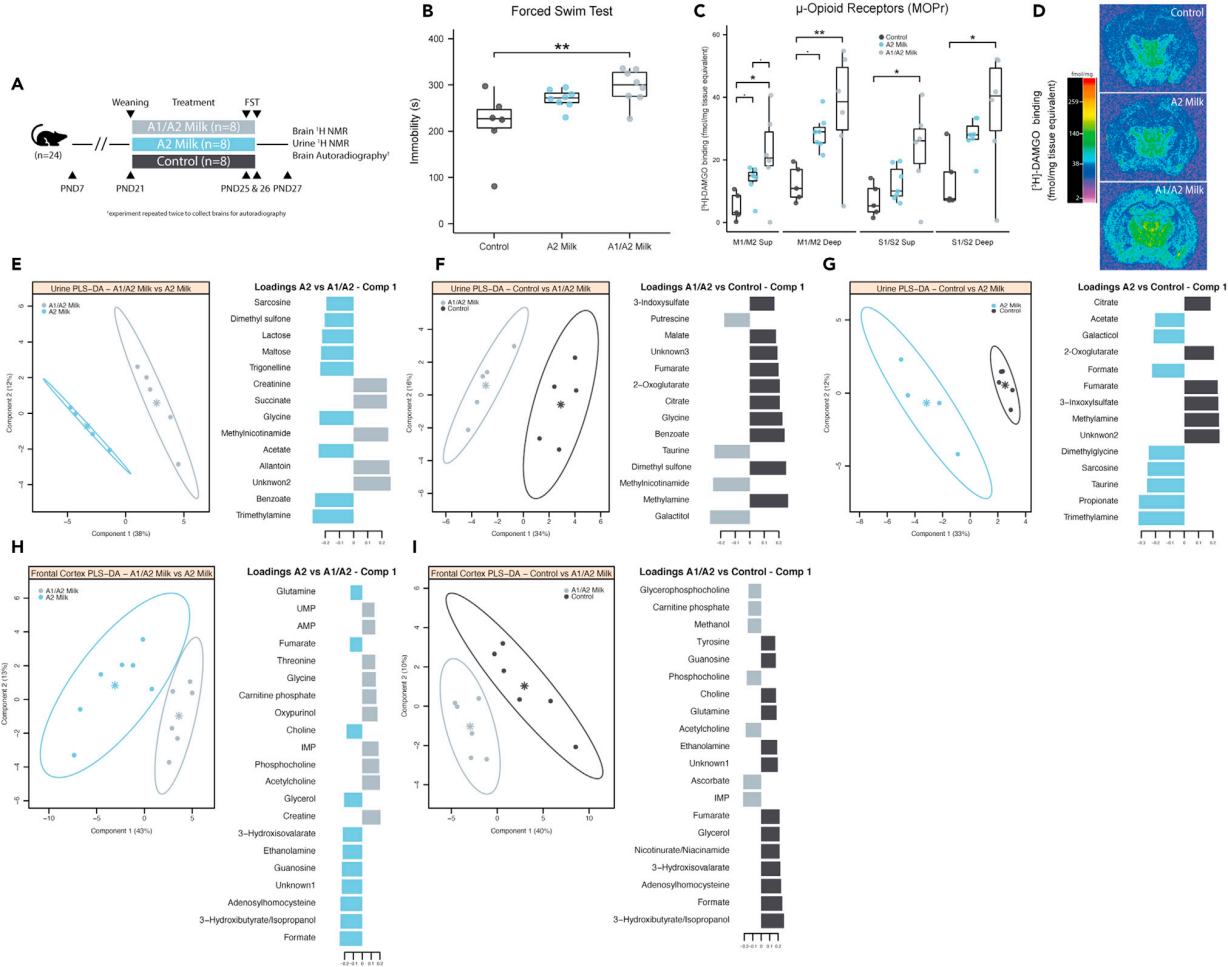
A1/A2 milk consumption beyond weaning age results in increased stress-induced immobility, indicative of depressive-like behavior, alterations in brain μ-opioid receptors (MOPr), and brain and urine biochemical profiles (A) Study design. Rats received at the experimental facility on PND7 and weaned on PND21. Divided in three groups, two received either A1/A2 milk or A2 milk while control group received only water from PND21 to PND26. The FST was carried out on PND25 (pre-test) and PND26 (test). Urines were collected for 24 hr on PND27, before sacrifice, in order to analyze them with ^1^H NMR spectroscopy. Brains were dissected, and the frontal cortex was analyzed with ^1^H NMR spectroscopy. Brains from different animal cohort were collected for receptor autoradiographic analysis. (B) Rats in the A1/A2 milk group, but not the A2 milk group, exhibited significantly increased immobility time in the FST compared to control (p = 0.009) (ANOVA followed by Tukey’s HSD post-hoc test). (C) Autoradiography of μ-opioid receptors (MOPr) in different brain regions. Animals fed A1/A2 milk showed significantly increased MOPr with respect to control in the motor cortex (M1/M2 Sup and M1/M2 Deep), somatosensory cortex (S1/S2 Sup and S1/S2 Deep), and hippocampus (ANOVA followed by Tukey’s HSD post-hoc test). (D) Computer-enhanced representative autoradiograms of adjacent coronal brain sections at the level of the thalamus (bregma −2.80 mm). μ-opioid receptors (MOPrs) labeled with [^3^H]-DAMGO (4nM) showed increased receptor binding across multiple brain regions in A1/A2 milk-treated animals compared to controls and A2 animals in some regions, including the deep layer of the motor cortex and hippocampus. The color bar illustrates a pseudocolor interpretation of black and white film images in fmol/mg tissue equivalent. (E**–**G**)** Pairwise PLS-DA of urine biochemical profiles and loadings of metabolites with VIP>1. Urine of control animals was characterized by a higher presence of methylamine, citrate, 2-oxoglutarate, fumarate, and 3-indoxysulfate compared to both the other two groups. Rats fed with A1/A2 milk excreted more methylnicotinamide compared to the other groups while rats fed with A2 milk acetate, sarcosine, and trimethylamine. (H**–**I**)** Pairwise PLS-DA of frontal cortex metabolic profiles and loadings of metabolites with VIP>1. Brain biochemical profiles of control and A2 milk animals were not significantly different. Rats fed with A1/A2 milk presented more acetylcholine, phosphocholine, and IMP in their frontal cortex compared to the other groups while the other presented more formate, adenosylhomocysteine, guanosine, ethanolamine, 3-hydroxyisovalarate, glycerol, choline, fumarate, and glutamine. Boxplots show first (lower) quartile, median, and third (upper) quartile. Significant and close to significant p values are represented as ⋅p < 0.1, ∗p < 0.05, ∗∗p < 0.01, ∗∗∗p < 0.001. Abbreviations: S1/S2 somatosensory cortex; M1/M2, motor cortex.

To investigate if the A1 and A2 β-casein variants are inducing different behavioral phenotypes and metabolic changes, as observed in the casein-rich vs casein-free study, we compared the consumption of commercialized bovine milk containing either a combination of A1 and A2 β-casein or exclusively A2 β-casein from PND21 to PND25 (Figure 3A). Consumption of commercial milk containing A1/A2 β-casein resulted in an increase in immobility time compared to the control group during the FST (p < 0.01; ANOVA followed by Tukey’s HSD post-hoc test) (Figure 3B). No significant difference in immobility time was observed between animals receiving A2 milk and control or commercial milk containing A1/A2 β-casein. Milk consumption per cage was comparable between groups, and no significant differences in weight gain were observed (Figure S5). Additionally, locomotor behavior assessed one hour before the FST on PND25 revealed no significant differences in motor activity between groups (Figure S6).

### Commercialized milk (A1/A2 β-casein) modulates MOPr and brain metabolic profiles

Quantitative autoradiographic binding of MOPr using [^3^H]-DAMGO was carried out on coronal sections across the whole brains of the animals. Commercial milk containing A1/A2 β-casein was found to induce higher levels of MOPr across several of brain regions. A significant increase in MOPr density was detected in the motor and somatosensory cortex, both superficial and deep, of rats exposed to commercial milk containing A1/A2 β-casein compared to control (p < 0.05; ANOVA followed by Tukey’s HSD post-hoc test) (Figures 3C and 3D). Notably, A2 β-casein milk exposure did not cause any significant changes in MOPr binding in these regions compared to control or commercial milk containing A1 β-casein. No significant differences were observed in any of the other analyzed regions (Figure S7).

To explore the potential role of these casein variants in modulating the neurobiochemical profiles of the animals, the frontal cortices collected on PND27 were analyzed using ^1^H NMR spectroscopy. PLS-DA models showed a clear separation of the A1/A2 milk group from the other two groups and also revealed that the biochemical profiles of A2 milk group and control were not significantly different, mirroring the MOPr binding results. Compared to both control (PLS-DA Q^2^Y = 0.38) and the A2 milk group (PLS-DA Q^2^Y = 0.64), the frontal cortices of animals fed with the commercial milk containing A1 β-casein contained a higher amount of phosphocholine, acetylcholine, IMP, and carnitine phosphate and a lower amount of 3-hydroxybutyrate, ethanolamine, choline, fumarate, guanosine, formate, glutamine, glycerol, 3-hydroxisovalarate, and adenosylhomocysteine (Figures 3H and 3I).

### Commercialized milk (A1/A2 β-casein) and A2 milk modulate urinary metabolic profiles

Urine of animals fed A1/A2 milk, A2 milk, or water were collected on PND27 and analyzed with ^1^H NMR spectroscopy. Pairwise PLS-DA models were generated to identify which metabolites were driving the separation between the groups. Urine of commercial milk containing A1/A2 β-casein group presented higher concentrations of creatinine, allantoin, succinate, and NMND and decreased abundance of acetate, trimethylamine (TMA), benzoate, glycine, trigonelline, maltose, lactose, dimethyl sulfone, and sarcosine compared to animals who received A2 milk (PLS-DA Q^2^Y = 0.61) (Figure 3E). Compared to control, commercial milk containing A1/A2 β-casein fed animal urine presented more galactitol, NMND, taurine, and putrescine but less methylamine, dimethyl sulfone, benzoate, glycine, citrate, 2-oxoglutarate, fumarate, malate, and 3-indoxysulfate (PLS-DA Q^2^Y = 0.73) (Figure 3F). Finally, the A2 milk group secreted more propionate, DMG, sarcosine, TMA, taurine, formate, galactitol, and acetate and less 2-oxoglutarate, citrate, methylamine, 3-indoxysulfate, and fumarate compared to the control group (PLS-DA Q^2^Y = 0.76) (Figure 3G).

### Urinary microbial metabolites correlate with brain metabolites

**Figure 4:**
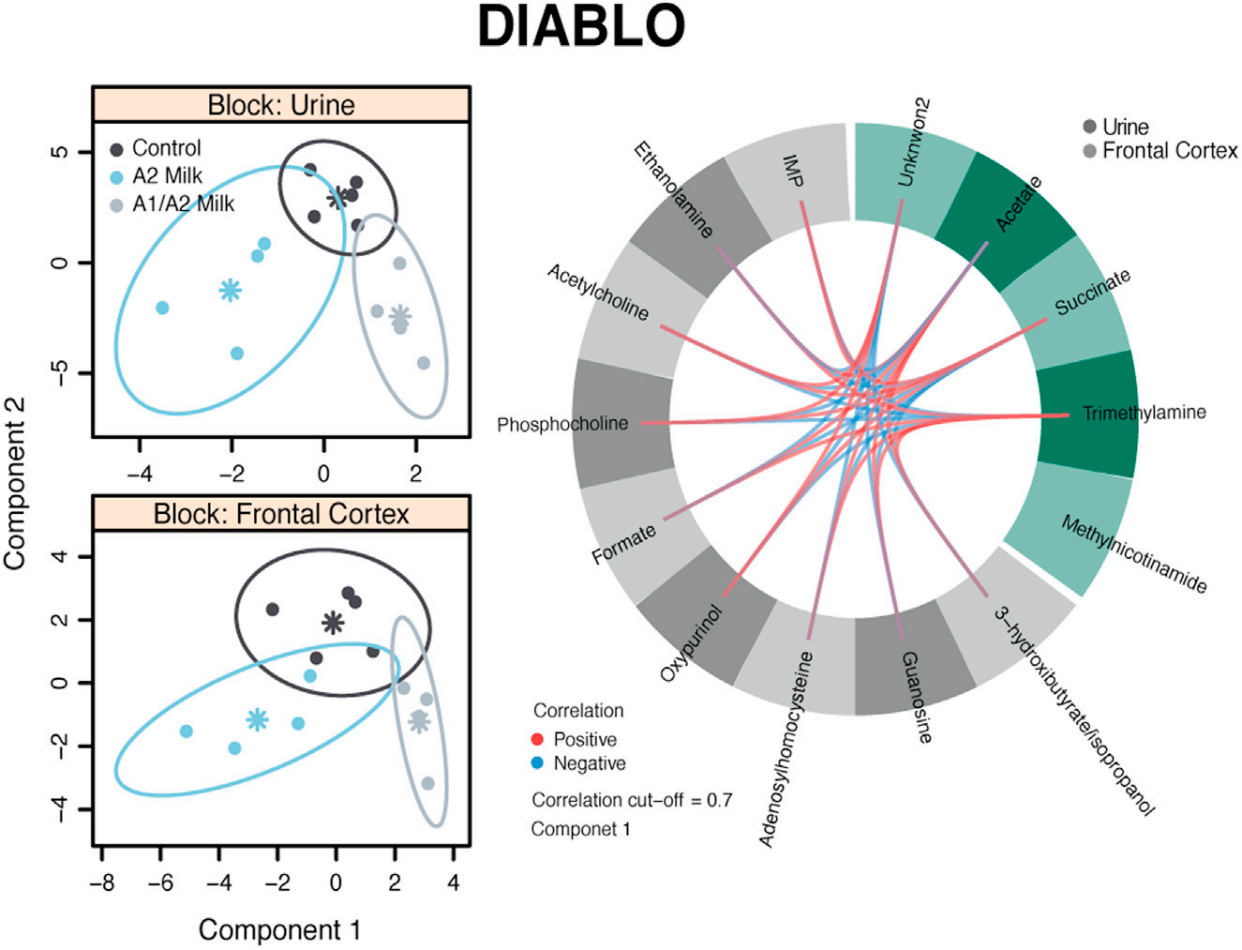
Integration of urine and frontal cortex biochemical profiles showed correlation between urinary microbial metabolites and several brain metabolites Projection of the latent structures with DIABLO showed separation of the different groups and high positive or negative correlation (r > 0.7) between several urine and brain metabolites on the first component. Urinary acetate and trimethylamine positively correlated with formate, adenosylhomocysteine, ethanolamine, and guanosine and also negatively correlated with acetylcholine, phosphocholine, oxypurinol, and IMP in the brain. Urinary succinate was positively correlated with IMP, acetylcholine, phosphocholine, and oxypurinol and negatively correlated with ethanolamine, formate, and adenosylhomocysteine in the brain.

DIABLO (Data Integration Analysis for Biomarker discovery using Latent variable approaches for Omics studies (Singh et al., 2019)) was used to integrate the urinary and brain metabolic profiles from control, commercial milk containing A1/A2 β-casein, and A2 β-casein milk groups and elucidate possible connections between endogenous and microbial metabolites in the urine and frontal cortex metabolism. A strong positive correlation (r > 0.7) was identified between acetate and trimethylamine, urinary metabolites derived from gut microbial activity, and formate, adenosylhomocysteine, ethanolamine, and guanosine present in the brain. These microbial products also negatively correlated with acetylcholine, phosphocholine, oxypurinol, and inosine monophosphate (IMP) in the frontal cortex. Urinary succinate, an endogenous host and microbial metabolite, was also found to be positively correlated with IMP, acetylcholine, phosphocholine, and oxypurinol and negatively correlated with ethanolamine, formate, and adenosylhomocysteine in the frontal cortex (Figure 4).

## Discussion

Here, we interrogated the impact of postweaning exposure to the casein component of milk and its two major variants A1 and A2 β-casein on behavioral, neurochemical, gut microbial, and metabolic endpoints. We demonstrate that postweaning exposure to casein-rich milk content (containing both A1 and A2 β-casein variants) or commercial milk, containing both A1 and A2 β-casein variants, enhances stress-induced immobility behavior during the FST, indicative of a depressive-like behavioral phenotype, and induces distinct neurochemical changes in receptor density and metabolomic profile. Neither casein-free milk nor that containing exclusively the A2 β-casein variant was able to induce such marked effects. This indicates that the A1 β-casein component of milk drives these observations.

In accordance with a previous study (Silva et al., 2015), a clear preference toward casein-rich milk compared to casein-free soy protein-based milk was detected with rats consuming more than double the amount of casein-rich milk compared to casein-free milk. As such, it is unclear if protein quantity rather than type (casein vs soy) may be the underlying cause of the observed immobility behavior. However, the lack of significant weight gain in the casein-rich group compared to casein-free and control groups, despite the increased milk consumption, suggests that rats are likely to have adapted their solid food and energy intake based on their protein need, which is a well-characterized behavior (Bensaïd et al., 2003). Several studies show that the amino acid composition of consumed protein, hence protein quality, can influence brain neurochemistry and behavior (Lasley and Thurmond, 1985; Chamberlain et al., 1987; Markus et al., 2000). Furthermore, ingestion of a soy protein diet in mice, before transitioning to a short photoperiod condition, was shown to result in lower immobility in the FST compared with mice provided a casein diet (Otsuka et al., 2015), further highlighting the impact of protein quality on FST immobility behavior. It is likely that the observed behavioral phenotype is the result of a combination of differences in the quantity and quality of protein, in this case casein. This is supported by our findings from the casein-spiked milk concentration response study. Here, a significant effect on immobility in the FST was observed only at a high casein dose of 20% w/w.

A significant decrease of MOPr binding was detected in the deep layer of the somatosensory cortex of rats with prolonged exposure to casein-rich milk compared to casein-free and control groups. This outcome may be mediated by peptide products derived by the proteolytic breakdown of casein, although this cannot be fully elucidated from this study. Milk caseins are a known source of exogenous opioid peptides, generated in the GI during digestion. These peptides can cross both the intestinal and the blood brain barriers to interact with opioid receptors in the brain (Zioudrou et al., 1979). The opioid activity found in mammalian milk is largely attributed to the presence of β-casein-derived BCM-5 and BCM-7, as well as α-casein-derived f90-96 and f90-95, which are all MOPr agonists (Brantl et al., 1981). Whether region-specific deficit in MOPr contributes to the generation of the observed behavioral phenotype cannot be ascertained from the current study but given the key role that MOPr plays in the modulation of mood (Lutz and Kieffer, 2013; Peciña et al., 2019) and the role of somatosensory cortex in emotional processing (Kang, 2018; Kropf et al., 2019), it is likely to be implicated. Changes to MOPr density were also observed in the nucleus accumbens of animals receiving both the casein-rich and casein-free milk compared to control animals, while casein-free milk alone decreased MOPr in the CPu, suggesting casein independent effects of milk on MOPr density. Soy protein, used to replace the casein component in the casein-free milk, was shown to release soymorphins, such as soymorphin-5, which are also specific ligands for MOPr (Ohinata et al., 2007). Hence, enhanced soymorphin generation in rats exposed to casein-free milk may explain the alterations observed in the CPu compared to control treated rats. Other mechanisms may also contribute to these observations. For example, Duraffourd et al. demonstrated that products from a high-protein diet can antagonize intestinal MOPs to activate a gut-brain neural pathway to induce satiety, indicating an antagonistic effect of protein quantity, irrespective of quality, on MOPr (Duraffourd et al., 2012). Besides the stimulatory effect of protein on satiety mechanism (Fromentin et al., 2012), there is evidence to suggest that a high protein diet can also modulate the brain reward system via poorly defined mechanisms (Journel et al., 2012).

OTr binding levels were downregulated in the basal lateral amygdala of animal fed casein-rich milk compared to both control and casein-free groups, indicating a casein-induced effect on OTr density. The role of the endogenous central oxytocinergic system as a regulator of stress-related behaviors and emotionality has been well established (Gigliucci et al., 2014; Zanos et al., 2014). Accumulating evidence suggests that oxytocin released in the amygdala, a key brain region involved in emotional processing (LeDoux, 2009), in response to stress, is responsible for the generation of passive stress-coping strategies (Yan et al., 2014). Dysregulation of the oxytocinergic system in the amygdala has been implicated in several stress-related neuropsychopathologies (Parker et al., 2010). For instance, low amygdala OTr levels have been consistently associated with increased anxiety (Yoshida et al., 2009). As a result, the effect of milk casein on the amygdala oxytocinergic system may contribute to the mechanism underlining the stress-induced immobility phenotype detected following casein milk exposure. No effect on OTr levels was observed in our previous study (Farshim et al., 2016), suggesting a more marked effect of casein on OTr density when delivered in a milk formula compared to prolonged breastfeeding. To our knowledge, this is the first study to demonstrate a marked and direct link between nutritional exposures in early life and the development of the brain OTr system, which may have lifelong implications in the regulation of stress reactivity (Bales and Perkeybile, 2012).

From urinary metabolic profiles, differences in the excretion of metabolites related to gut microbial activity were observed between rats receiving either casein-rich or casein-free milk. Animals consuming casein-rich milk excreted greater amounts of hippurate, a microbial-mammalian co-metabolite arising from the bacterial breakdown of plant phenols to produce benzoate, which is subsequently conjugated with glycine in the liver to produce hippurate (Temellini et al., 1993). This metabolite was significantly correlated with increased animal immobility time in our study. Variation in hippurate excretion may indicate an increase in the activity of certain bacterial species with casein intake, increasing the production of benzoate and depleting the availability of glycine, an excitatory amino acid found to be lower in the plasma of patients with major depression (Altamura et al., 1995). Consistently, the casein-rich group excreted lower amounts of glycine compared to control animals, as well as lower amounts of dimethylglycine and sarcosine (methyl-glycine). Such changes may reflect compensatory strategies to increase glycine availability. Interestingly, lower amounts of dimethylglycine have been documented in patients with depression (Zheng et al., 2013), and sarcosine intake has been shown to improve depressive-like behavior (Chen et al., 2017).

A significant increase in the abundance of bacteria in the *C. histolyticum* group was noted in the cecum and colon of rats consuming casein-rich milk compared to those fed casein-free milk, following the FST. Interestingly, hippurate excretion has previously been linked to increased abundances of the *Clostridium* genus in the gut (Pallister et al., 2017). Differences in protein quantity and quality, in combination with stress, may underlie these observations and modify the production of metabolites from the host genome and microbiome. As with behavior and neurochemistry, gut microbial composition and their respective metabolic products are known to be influenced by protein quantity and quality (Zhao et al., 2018). For example, greater amounts of undigested protein have been shown to lead to an increase of pathogenic microorganisms associated with a higher risk of metabolic diseases (Zhao et al., 2018). Moreover, the abundance of *Akkermansia* and *Bifidobacterium* has been shown to increase in mice supplemented with branched-chain amino acids (Yang et al., 2016). These findings and the observation that different amino acids affect host and bacterial metabolism differently (Madsen et al., 2017) suggest that the microbial and metabolic differences reported here are driven by differences in protein quality (i.e. amino acid composition of casein vs soy), albeit at higher levels in the casein-rich group.

Despite the two milk diets being carefully nutritionally matched, there were subtle differences in the compositional breakdown of the macronutrients. For example, the fat source of casein-rich milk came from vegetables and fat mix, whereas for casein-free milk, it came from maize and soy oil, indicating differences in the concentrations of saturated and monounsaturated and polyunsaturated fatty acids in the two milks. Given that the casein-rich group consumed significantly more milk than the casein-free group, it is likely that these rats consumed higher amount of fats. However, no significant differences were noted in weight gain between the study groups, suggesting that this difference in nutrient intake had a minimal effect on metabolism and body weight. A study investigating the effects of different dietary oils on FST behavior failed to find any significant differences in immobility behavior between the different groups (Kato, 2015), suggesting that quality of fat may not influence immobility behavior. It is plausible that differences in quantity and quality of fat consumed may have an impact on MOPr and OTR density in the brain. A recent study by Deol et al. demonstrated a profound dysregulation of hypothalamic gene expression in mice fed soy oil-containing diet compared to control and coconut oil-containing diet with different proportions of fatty acid composition (Deol et al., 2020). An increase in oxytocin expression and a decrease of oxytocin immunoreactivity were detected in the hypothalamus of mice fed diet containing soybean oil compared to mice fed control or coconut oil-containing diet, clearly demonstrating that quality of fat consumption impacts on brain neurochemistry. Although in the current study, no effect of milk (either casein rich or casein free) was detected on OTR density in the hypothalamus and OTR density was not influenced by soya oil-containing casein-free diet, the possibility that changes in neurochemistry may be influenced by consumption of fats of different fatty acid composition warrants future investigation.

Consistent with casein-rich milk, exposure to commercial milk containing A1/A2 β-casein for 5 days after weaning was also found to result in increased stress-induced immobility behavior during the FST, suggestive of a depressive-like phenotype. Such observations were not seen in animals fed bovine A2 β-casein milk postweaning or control animals, suggesting that the bioactive compound arising from the breakdown of A1 β-casein, BCM-7, may be at least partly responsible for this behavioral phenotype. Unlike the casein-rich milk fed group, rats consumed the same amount of commercial bovine A1/A2 β-casein (A1/A2 group) and A2 β-casein (A2 group) containing milks. Given that the macronutrient composition of the two bovine milks were closely matched with only the β-casein variant differing, it is highly unlikely that any behavioral, neurochemical, and metabolic differences observed following consumption of the two milks is caused by differences in quantity or quality of macronutrients (e.g. fats, proteins). Intriguingly, exposure to commercial milk containing A1/A2 β-casein was found to induce a significant region-specific upregulation of MOPr in the brain. This was not seen in the rats receiving the A2 β-casein, indicating that BCM-7 may be at least partly driving this MOPr upregulation in the motor and somatosensory cortex of the brain. Consistently, previous work has demonstrated that incubation of peripheral blood mononuclear cells from children with atopic dermatitis with peptide extracts from cow milk containing BCM-7 increased MOPr gene expression (Fiedorowicz et al., 2014). While it is unclear if the increase in MOPr contributes to the observed immobility phenotype, dysregulation of MOPr function has been implicated in several psychopathologies including depression, autism spectrum disorder, and schizophrenia (Scarr et al., 2012; Pellissier et al., 2018).

Surprisingly, the direction of MOPr density alterations following exposure to commercial milk containing A1/A2 β-casein in the somatosensory cortex is opposite of that detected following casein-rich milk exposure, despite both containing A1 β-casein variant. Nonetheless, there are subtle differences in micronutrient composition (e.g. fat, protein) of the two milks which may have affected neurochemistry as discussed above. There was a clear preference for the commercial milk containing A1/A2 β-casein compared to casein-rich milk with rats consuming on average twice as much A1/A2 milk than casein-rich milk (161.46 g vs 83.22 g) from PND21 to PND25. As such, differences in the quantity of macronutrients, including protein, could also be driving this divergent effect on MOPr density in the somatosensory cortex. This effect is highly unlikely to be driven by milk A1 β-casein as we would have expected an upregulation, rather than a downregulation of MOPr in the somatosensory and motor cortex of casein-rich rats, consistent with the effect demonstrated in rats consuming commercial milk containing A1/A2 β-casein. It is indeed plausible that the effect of A1 β-casein on MOPr expression may reflect an agonistic activity of its breakdown peptide BCM7 on the receptor. Regardless of the differential effect of casein-rich and commercial milk containing A1/A2 β-casein on MOPr density, our findings suggest that intake of milk containing A1 β-casein but not milk containing solely A2 β-casein variant or casein-free milk postweaning negatively affects immobility behavior and induces brain-specific changes in MOPr density.

From the biochemical analysis of the frontal cortex, a region involved in cognition, decision-making, and emotional regulation, commercial milk containing A1 β-casein was found to have a substantial impact on the metabolic landscape of the brain compared to both A2 milk and control groups, which had similar profiles. This emphasizes the importance of early-life nutritional exposures on neurobiochemistry. Several choline-related metabolites were altered in response to commercial milk containing A1 β-casein. Lower amounts of choline and ethanolamine and higher amounts of phosphocholine and acetylcholine were observed in the frontal cortex of animals fed commercial milk containing A1 β-casein compared to both control and A2 β-casein groups. Choline can be synthesized endogenously, but this process is insufficient to meet the demands of the body. As such, choline is an essential nutrient that is mainly obtained from the diet. This metabolite is necessary for many functions, including cell membrane structural integrity, cell signaling, and neurotransmission (Sanders and Zeisel, 2007). Considering these diverse roles, disruptions in choline metabolism, especially during early life, may have important consequences for brain development and function (Zeisel, 2006). Choline is also a major source of methyl donors, important for epigenetic regulation (Zeisel, 2006). DNA methylation is a key process in the developing brain, with methylation changes in specific genes linked to depression (Duan and Lu, 2020). During the methylation process, *S*-adenosylmethionine is converted to *S-*adenosylhomocysteine as it donates a methyl group to the DNA. In this study, *S-*adenosylhomocysteine was present in lower amounts in the commercial milk containing the A1/A2 β-casein group, suggesting hypomethylation in the frontal cortex of these animals. This is in line with a previously reported decrease in the *S*-adenosylmethionine/*S*-adenosylhomocysteine ratio and DNA methylation states induced by BCM-7 exposure in differentiating neuronal stem cells (Trivedi et al., 2016).

Integrative analysis identified associations between gut microbial derived metabolites in the urine and several metabolites in the brain which were altered by the type of β-casein variant consumed. This highlights the variety of molecules that contribute to the communication between the gut microbiota and the brain and their potential to be modified by dietary exposures. For example, trimethylamine, a microbial metabolite of dietary choline, was negatively correlated with the membrane component phosphocholine and the neurotransmitter acetylcholine. This suggests that gut microbial activity can modulate the availability of choline with downstream consequences for the abundance of these important neurological molecules. The short-chain fatty acid acetate was also positively associated with formate, *S*-adenosylhomocysteine, and guanosine in the brain and negatively correlated with oxypurinol, phosphocholine, and acetylcholine. Gut-derived acetate can translocate to the brain and can affect histone acetylation and thus the epigenetic landscape of the brain, influencing behavior (Mews et al., 2019; Osman et al., 2020). Decreased urinary excretion of acetate was noted in animals fed A1 β-casein compared to animals who received A2 β-casein milk. A study in mice corroborates our findings and shows that milk containing only A2 β-casein results in higher fecal short-chain fatty acids concentrations compared to milk containing a mixture of A1 and A2 β-casein (Guantario et al., 2020).

The enzyme responsible for the hydrolysis of A1 β-casein to produce BCM-7 is dipeptidyl peptidase-4 (DPP-4). This enzyme is typically found in epithelial and endothelial cells, in a soluble form in the blood, and also anchored to cell membranes of bacteria present in the gut (Da Silva, 2019). Its activity has been linked with behavioral disorders in both rats and humans (Canneva et al., 2015; Kamble et al., 2016). DPP-4 has a spectrum of actions beyond its role as a proteolytic enzyme. It also has a well-established role in glucose regulation where it regulates the biological activity of the incretin hormone, glucogen-like peptide-1, helping to maintain normal glucose homeostasis (Deacon, 2019). To this end, A1/A2 milk consumption resulted in the disruption of several brain metabolites involved in energy homeostasis including IMP and adenosime monophosphate (AMP), which are breakdown products of ATP, and 3-hydroxybutyrate, a ketone body synthesized in the liver and used as an energy source by the brain when blood glucose is low. Compared to control animals, those fed with commercial milk containing A1/A2 β-casein or A2 milk excreted lower amounts of metabolites involved in the tricarboxylic acid (TCA) cycle such as citrate and 2-oxoglutarate. This is consistent with findings from Farshim et al. who also reported lower levels of TCA cycle-related metabolites in the urine of non-weaned animals compared to normally weaned counterparts (Farshim et al., 2016). Moreover, lower 2-oxoglutarate and citrate levels have previously been reported in a rat model of depression (Zheng et al., 2011). Together, these findings link β-casein exposure postweaning to a dysregulation in energy homeostasis.

In conclusion, the data presented here indicate that dietary manipulations around weaning age in the form of consumption of milk containing both A1/A2 β-casein but not A2 variant alone have important implications for brain development and emotional behavior via a possible gut-brain axis-mediated mechanism. It is evident that, intake of milk containing the A1 β casein variant in early postnatal life when both brain and microbiome development are sensitive to environmental factors negatively affects emotional behavior and induces concomitant brain region-specific neurochemical and metabolic changes alongside alterations to gut-derived metabolic profile. As milk is the major dietary source in infants, the conclusions of this study emphasize the need to assess the translation relevance of this effect and, if reproduced, inform the importance of selecting A1 β casein-free milk and dairy products for consumption during this sensitive postweaning developmental period.

### Limitations of the study

Limitations of this study include differences in the composition of the utilized milks. As mentioned in the discussion, differences in lipid profiles between the various milks may have had an influence on brain receptor densities. Additionally, milk consumption was recorded per cage and not per animal as individually housing animals is a stressor, given the social nature of rodents. In future, methods to detect individual animal milk intake should be implemented. Furthermore, the observed altered phenotypes were attributed to A1 β-casein and ultimately to its breakdown product BCM-7. Nevertheless, BCM-7 was not directly measured. In the future, collection of blood and fecal content within two hours of milk consumption should be taken into consideration in order to investigate the presence of BCM-7. Our results suggest that A1 β-casein and BCM-7 play a fundamental role in neurological and behavioral development and provide information that will inform the development of future more detailed mechanistic studies to investigate this relationship.

## STAR★Methods

### Key resources table

**Table:** 

REAGENT or RESOURCE	SOURCE	IDENTIFIER
**Chemicals, peptides, and recombinant proteins**		

TSP	Sigma-Aldrich	24493-21-8
D_2_O	Sigma-Aldrich	7789-20-0
KH_2_PO_4_	Sigma-Aldrich	7778-77-0
[^125^I]-OVTA	Perkin Elmer	NEX254010UC
[^3^H]-DAMGO	Sigma-Aldrich	100929-53-1
(Thr4, Gly7)-oxytocin	BACHEM	4013837.0005

**Deposited data**		

Processed Data	This Manuscript	https://doi.org/10.5281/zenodo.5174852

**Experimental models: Organisms/strains**		

Wistar Albino Rats	Charles River UK Limited	003

**Oligonucleotides**		

5′-CCAATGTGGGGGACCTT	Sigma-Aldrich	BIF164 Bifidobacterium spp.
5′-TTATGCGGTATTAATCYCCTTT	Sigma-Aldrich	CHIS 150 Clostridium histolyticum group.
5′-GGTATTAGCAYCTGTTTCCA	Sigma-Aldrich	LAB158 Lactobacillus – Enterococcus
5′-GCTGCCTCCCGTAGGAGT	Sigma-Aldrich	EUB338I (total domain bacteria I)
5′-GCAGCCACCCGTAGGTGT	Sigma-Aldrich	EUB338II (total domain bacteria II)
5′-GCTGCCACCCGTAGGTGT	Sigma-Aldrich	EUB338III (total domain bacteria III)

**Software and algorithms**		

R (version 4.0.2)	The R Foundation for Statistical Computing	https://cran.r-project.org/
R Scripts	This manuscript	https://doi.org/10.5281/zenodo.5174852
TOPSIN 3.2	BRUKER	https://www.bruker.com/en.html
MATLAB 2017a	MathWorks	https://uk.mathworks.com/products/matlab.html

### Resource availability

#### Lead contact

Further information and requests for resources and reagents should be directed to and will be fulfilled by the lead contact, Jonathan Swann (j.swann@soton.ac.uk).

#### Materials availability

This study did not generate new unique reagents.

### Experimental model and subject details

Male, Wistar albino rats (Charles River UK Limited, Kent) were used in all experiments. Two experimental groups were generated, the Casein experimental group which was conducted at the University of Surrey Experimental Animal Unit and the A1/A2 milk experimental group which was conducted at St. George’s University London Biological Research Facility. For each study, three groups of animals each containing eight male pups plus their cross-fostered mother were ordered to arrive at PND7, where day of birth is PND0. These animals were maintained in litters of 8 pups with their mothers at all times. Pups were left to acclimatize to their new environment for 7 days (until PND14), after which point, they were handled and weighed daily by the experimenter. All groups were placed in a 12-hour light-dark cycle (lights on 6AM - 6PM) with free access to a standard rat chow diet (B & K Universal Ltd for the casein-rich study and SDS for the A1/A2 study. See Table S1 for nutritional content of the respective diets). All groups were kept at a constant temperature of 22°C ± 1°C, humidity of 45-55%. In the Casein study, all groups were weaned on PND21 and had free access to rat chow and water. The control group was not provided with any milk post-weaning, the casein-rich group was provided with casein-rich milk (Welpi; Pet Life International Ltd, Bury St. Edmunds UK, casein derived from cow’s milk) from PND21 until PND25. Casein-rich milk was made up by mixing one level scoop of powder (5 g containing 20% w/w casein) with two level scoops of water (20 g) i.e. 1 part powder to 2 parts water by volume to make 25 mL of liquid Welpi. There was no supplementation of external source of casein. The casein-free group was provided with milk in which the casein component was removed and replaced with soy protein (20% w/w of milk powder) (Special Diet services, Cambridge, UK). Casein-free milk was made up in an identical way as casein-rich milk. For the casein dose response study, identical casein-free formula utilized in the aforementioned casein study was spiked with 10% or 20% w/w casein (Sigma, Poole, UK) in milk powder and provided to weaned pups from PND21. A control group was included which consumed unspiked casein-free milk. Similarly, in the A1/A2 milk study all animals were weaned on PND21 and had free access to rat chow and water. This study consisted of a control group, a group provided with conventional cow’s milk containing a mixture of A1/A2 β-casein (Commercialized whole milk) and a group provided with milk containing exclusively A2 β-casein (a2 Milk company) from PND21 to PND25. Nutritional content of the various types of milks is reported in Table S2. All experimental procedures were conducted in accordance with the U.K. Animal Scientific Procedures Act (1986).

### Method details

#### Forced swim test

All behavioral tests were carried out between 9 AM and 5 PM. The FST, a two day test, was used to assess the total time animals spent immobile not attempting to escape from water, a measure which is sensitive to antidepressant in rats, and it was carried out as originally described by Porsolt et al. (1978). Briefly, animals were left to acclimatize to the behavioral room, in their home cages, for 1 hrhour prior to testing. On the first day of testing (PND25), animals were exposed to a 15 min “pre-test” followed 24 hours later by a 6 minutes “test” session. Each rat was individually placed in a glass tank containing tap water maintained at a temp of 23°C ± 1°C (Diameter: 170 nm x Height: 270 cm Fisher Scientific UK). Immediately after each session, the animals were dried using a paper towel and then placed in a warm (30°C ± 2°C) fanned recovery chamber for 15 minutes to dry before being returned to home cages. The “pre-test” and “test” sessions were recorded using a video camera placed in front of the glass beakers and scored by 3 independent observers who were blind to the identity of the treatment groups of each animal. The total time spent immobile during “test” day on PND26 was quantified from the videos. This was assessed as >2.0 seconds making only movements necessary to keep head over water. Animals with 0 seconds spent immobile during the test were excluded from the study and further analysis. All behavioral tests were carried out under low illuminations levels (∼40 lux).

#### Locomotor test

Locomotor activity of each animal in the A1/A2 milk study was assessed for the duration of 20 minutes 1 hour prior the “pre-test” of the FST (PND25). Animals were firstly brought into the locomotor testing room in their home cages and left to acclimatize 1 hour prior to testing. Animals were then individually placed in locomotor chambers (40 cm x 20 cm x 20 cm; Linton Instrumentation, Norfolk, UK). Each chamber consisted of 2 rows of 16 photocells located at right angles to each other projecting horizontal infrared beams 2.5 cm apart and 6 cm above the cage floor to measure horizontal and vertical activity, respectively. Activity was recorded as the number of sequential beam breaks in 5-minute bins.

#### Sample collection

Animals were individually housed in metabolic cages for 24 hours on PND26 after the FST. Urine samples gathered from the metabolic cages were collected between 10:00 am and 11:00 am on PND27 and stored at -80°C. On PND27, animals were scarified through cervical dislocation. Intact brains were dissected and rapidly frozen in isopentane at −20 °C for 30 seconds and then stored at −80 °C until quantitative receptor autoradiography or ^1^H NMR spectroscopy analysis. The carcasses were transferred into a fume hood and duodenum, jejunum, ileum, cecum and colon were dissected. Contents from these regions were weighed (∼70–200 mg) into sterile 2 ml Eppendorf tubes and kept over ice.

#### Fluorescence *in situ* hybridization

Gastrointestinal content from five different gut regions (duodenum, jejunum, ileum, caecum and colon) were collected in the Casein study and diluted 1:10 (w/v) in PBS (0.1 M, pH 7.0; Oxoid). Tubes were centrifuged at 2000 g for 2 minutes and the supernatant collected into a 2 mL Eppendorf tube. This process was repeated twice, with each supernatant being added to the same 2 mL tube. The resulting supernatant was centrifuged at 13000 g for 5 minutes and the pellet resuspended in 750 μL of PBS and then fixed with using 4% (w/v) paraformaldehyde solution (2.25 mL, Sigma) for 4 hours at 4°C. Following paraformaldehyde fixation, cells were washed twice in PBS by centrifugation at 13000 g for 5 minutes, resuspended in 600 μL PBS/ethanol mixture (1:1) and stored at −20 °C at least 4 hours prior to hybridization. Intestinal bacterial population was assessed by FISH analysis using a collection of 5′Cy3-labeled 16S rRNA oligonucleotide probes (Sigma Aldrich, Dorset, UK). Oligonucleotide probes targeting *Bifidobacterium* spp. (5′-CCAATGTGGGGGACCTT) Bif164; *Clostridium histolyticum* group (*Clostridium* clusters I and II) (5′-TTATGCGGTATTAATCYCCTTT), Chis150; *Lactobacilli/Enterococci* spp. (5′-GGTATTAGCAYCTGTTTCCA) Lab158 were used to investigate specific functional group of the gut microbial community (Alm et al., 1996). For total bacterial cells count, a mixture of different probes (EUB338I/II/III; 5′-GCTGCCTCCCGTAGGAGT, 5′-GCAGCCACCCGTAGGTGT and 5′-GCTGCCACCCGTAGGTGT) was used. Slides were enumerated using an EPI-fluorescence Eclipse E400 Nikon microscope (Nikon, U.K., Kingston-upon-Thames, U.K). Fifteen random microscopic fields of view were counted per assay and used to calculate the number of cells per gram of original sample.

#### Quantitative OTr and MOPr autoradiography

Adjacent 20 μm coronal sections from animals of the Casein study and A1/A2 study were cut at 400 μm intervals at −21 °C using a cryostat apparatus (Zeiss Microm 505E, Hertfordshire, U.K.) and thaw-mounted onto gelatin-coated ice-cold microscope slides to define receptor binding levels from fore- to hind-brain regions. Brain slides were stored at −20 °C in airtight containers containing a layer of anhydrous calcium sulfate (Dreirite-BDH Chemicals, Dorset, U.K.) until used. OTr and MOPr autoradiography were performed in accordance with previously described methods (Zanos et al., 2014) with minor modifications. Briefly, sections were rinsed twice for 10 minutes for OTr binding and 20 mins for MOPr binding in a pre-incubation buffer solution (50 mM Tris-HCl, pH 7.4) at room temperature to remove endogenous oxytocin and opioids. For OTr, the total binding was determined by incubating the sections with 50 pM [^125^I]-Ornithine vasotocin analog [d(CH_2_)5[Tyr(Me)^2^,Thr^4^,Orn^8^,[^125^I]Tyr^9^-NH_2_]-vasotocin] ([^125^I]-OVTA) (Perkin Elmer, Boston, MA) and for MOPr [^3^H]-DAMGO (4nM) (Sigma-Aldrich, Poole, UK) in the same incubation buffer medium. For OTr, adjacent sections were incubated with [^125^I]-OVTA (50 pM) for 60 minutes in the presence of 50 μM of OTr ligand, (Thr4, Gly7)-oxytocin (Bachem, Germany), to determine non-specific binding (NSB). For MOPr, NSB was determined by incubating adjacent sections with 1 μM naloxone for 1 hour. Following the radioligand binding period, slides were rinsed 3 times for 5 minutes in ice cold rinse buffer (50 mM Tris-HCl, 10 mM MgCl2, pH 7.4) followed by a 30-minute wash in the ice-cold rinse buffer and a subsequent 2 second wash in ice-cold distilled water. Slides were then dried under a stream of cool air for 2 hours and stored in sealed containers with anhydrous calcium sulfate (Drierite BDH Chemicals, Dorset, U.K) for 2 days. Slides were opposed to Kodak MR-1 films (Sigma-Aldrich, U.K.) in hypercassettes with autoradiographic [^14^C] microscales of known radioactive concentration (GE Healthcare Life Sciences, Amersham, U.K.) for 3 days for the oxytocin binding and with autoradiographic [^3^H] microscales for 10 weeks for MOPr binding. Films were developed using 50% Kodak D19 developer (Sigma-Aldrich, Poole, U.K.). Quantitative analysis of autoradiographic films was carried out using MCID image analyzer (Image Research, Ontario, Canada) as previously described (Kitchen et al., 1997). Optical density values were quantified from the [^14^C]-microscale for OTr analysis and a [^3^H] microscale for MOPr analysis (GE Healthcare, U.K.) which were entered with corresponding radioactivity values into a calibration table on MCID and the relationship between radioactivity and optical density was subsequently determined. Analysis of brain structures were carried out by reference to the rat brain atlas of Paxinos and Watson (1998). The following structures were analyzed by sampling 5–8 times with a box tool: cortex (8 × 8 mm), olfactory tubercle (6 × 6 mm) and hippocampus (5 × 5 mm). While central brain regions such as hypothalamus and thalamus were analyzed by freehand drawing. Measures were taken from both the right and left hemispheres, thus representing a duplicate determination for each region. Sections for all treatment groups were processed in parallel and opposed to the same film at the same time.

#### ^1^H nuclear magnetic resonance (NMR) spectroscopy

Urines were defrosted and vortexed. Samples were prepared adding 200 μL of phosphate buffer (100% D_2_O, 1.5 M KH_2_PO_4_, 1 mM TSP and 2 mM sodium azide) to 400 μL of urine. After centrifugation at 13000 g for 10 minutes at 4°C, 600 μL of supernatant were transferred into 5 mm NMR tubes.

Brains were defrosted and ∼80 mg of frontal cortex were weighted into hominization tubes. Ten zirconium beads and 300 μL of a 2:1 solution of clorophorm/methanol (CHCl3/MeOH) were added to each tube. Vials were constantly kept on ice until homogenization. A Precellys 24 homogenizer (Bertin Instruments) was used to run the samples twice at 5500 rpm in a 1 minute program (2x20 sec homogenization with an interval of 20 sec in between). To each homogenized sample, 300 μL of demineralized water were added and then centrifuged at 13000 g for 10 minutes at 4°C. The aqueous phase was collected in a separate tube while the organic phase was discarded. The extraction step was repeated twice to collect a total of 600 μL of aqueous phase. Tubes were left to dry overnight in a vacuum condenser at room temperature. Samples were reconstituted with 700 μL of phosphate buffer (100% D_2_O, 1.5 M KH_2_PO_4_, 1 mM TSP and 2 mM sodium azide) on the day of the experiment, vortexed and centrifugated at 13000 g for 10 minutes at 4°C. Finally, 600 μL of supernatant were transferred into 5 mm NMR tubes.

For both urine and frontal cortex samples, tubes were loaded into a refrigerated (4°C) SampleJet robot by Bruker and standard NOESY (1D NMR) experiments were run in automation at 300 °K on a Bruker 600 MHz UltraShield spectrometer (Bruker Biospin, Karlsruhe, Germany). A standard one-dimensional pulse sequence with saturation of the water resonance was applied (RD-90°-t_1_-90°-t_m_-90°-acquire FID, with RD set at 2 sec and t_m_ at 100 ms). For each spectrum 8 dummy scans, followed by 64 scans with 32K data points and a spectral width of 20,000 Hz were acquired.

#### ^1^H NMR data processing and analysis

Spectra were manually corrected for phase and baseline distortion to the TSP singlet (δ 0.00) before data acquisition on TOPSPIN 3.2 (Bruker, Germany). The obtained spectra were digitized on MATLAB 2017a using IMPaCTS (https://github.com/csmsoftware/IMPaCTS). Unwanted regions of the spectra were removed: TSP (-0.2 to 0.2 ppm), water (4.7 to 4.9 ppm) and the urea region (5.48 to 6.23 ppm) in urine samples. Peaks were manually aligned through recursive sample-wise peak alignment (RSPA) present in IMPaCTS and normalized to median fold change for urine and to the weight of the sample for brains. Principal component analysis (PCA) with pareto scaling was used for preliminary unsupervised analysis and outlier identification. Strong outliers, when present, were removed from further analysis. Peak were annotated using in-house database, Chenomx (Chenomx Inc, Edmonton, Canada), HMDB (http://www.hmdb.ca/) and statistical total correlation spectroscopy (STOCSY) to identify peaks belonging to the same metabolite. Annotated metabolites and unknown peaks were integrated from the spectra to recover the highest possible number of metabolites to be used in the downstream analysis.

### Quantification and statistical analysis

All statistical analyses were performed on R version 4.0.2 (R Foundation for Statistical Computing, Vienna, Austria). ANOVA, followed by Tukey HSD pos-hoc test, was used to compare the mean immobility time between groups in the different studies and to evaluate differences in MOPr and OTr binding for the brain autoradiography experiments. When ANOVA assumptions were not met, Kruskal-Wallis, followed by pairwise Wilcoxon tests and Benjamini-Hochberg (BH) correction, was used. When ANOVA resulted significant both significant (p < 0.05) and close to significant (p < 0.01) pairwise comparisons resulted from Tukey HSD pos-hoc test were reported. Wilcoxon test, followed by BH correction, was used to compare the mean of log_10_ counts in the FISH obtained data, reported significance for p < 0.05. The `ropls` and `mixOmics` packages were used to generate the PCA and PLS-DA models of the integrated metabolites, which were previously log_10_ transformed. The `ropls` package was used to evaluate the significance of the PLS-DA models using a 7-fold cross validation and 999 permutations. Metabolites with variable importance projection (VIP) > 1 were considered relevant for the models and retained in the loading plots of the PLS-DA models. The metabolic correlation network was generated calculating Spearman correlation between metabolites and immobility time using the `Hmisc` package. P values were corrected for false discovering rate (BH) and only correlations with p adjusted < 0.05 were kept and visualized in the network. The final network was generated using package `igraph` and then exported to Cytoscape 3.8.2 for further refinement. Urine and brain metabolic profiles from the A1/A2 study were integrated using the DIABLO function from the `mixOmics` package. First, the correlation between the two omics-blocks was calculated using the PLS function in `mixOmics`. The obtained correlation value was then used in the DIABLO design matrix to select highly correlated and discriminatory variables. Circos plot was generated utilizing only the first component of the obtained model and displaying highly correlated pairs (r > 0.7).

## Data Availability

• Data utilized for the analysis and to produce all the figures is available on GitHub (https://github.com/simonezuffa/Casein_Manuscript) and deposited at Zenodo. DOI is listed in the key resource table. Raw NMR spectra will be shared by the lead contact upon request. • All original code has been deposited at Zenodo and is publicly available as of the date of publication. DOI is listed in the key resources table. • Any additional information required to reanalyze the data reported in this paper is available from the lead contact upon request.
