# Supplementary material for: Post-weaning A1/A2 β-casein milk intake modulates depressive-like behavior, brain μ-opioid receptors, and the metabolome of rats
Source: iScience. 2021 Aug 28;24(9):103048. doi: 10.1016/j.isci.2021.103048 (PMC8450247; doi:10.1016/j.isci.2021.103048)
Supplement: Document S1. Figures S1–S7 — and Tables S1 and S2 [file mmc1.pdf]

iScience, Volume *24*

Supplemental information

Post-weaning A1/A2 β-casein milk intake modulates depressive-like behavior, brain μ-opioid receptors, and the metabolome of rats

Aya Osman, Simone Zuffa, Gemma Walton, Elizabeth Fagbodun, Panos Zanos, Polymnia Georgiou, Ian Kitchen, Jonathan Swann, and Alexis Bailey

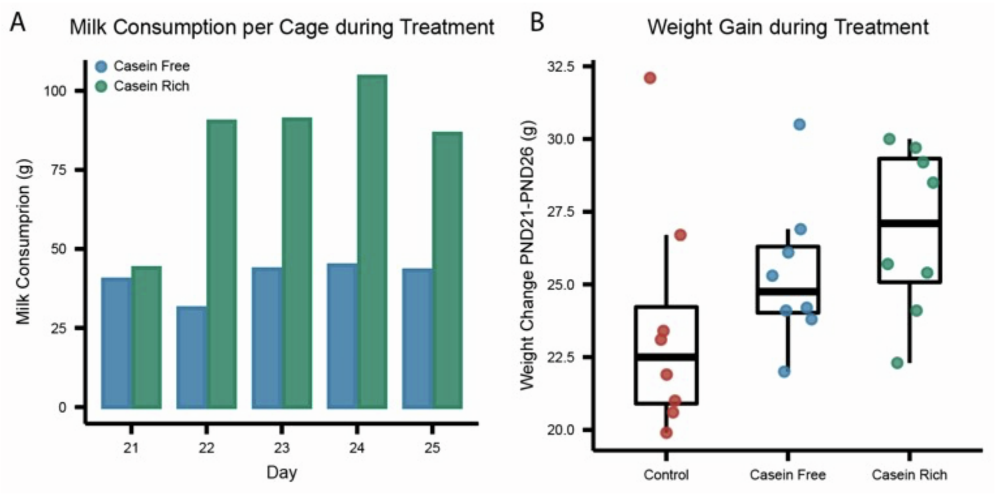

Figure S1. Milk consumption and weight gain during intervention in Casein Study, related to Figure 1
A) Animals consumed more casein-rich milk compared to casein-free milk per cage. B) Higher milk consumption did not result in significant differences in weight gain between groups during treatment. ANOVA followed by Tukey's HSD post-hoc test.

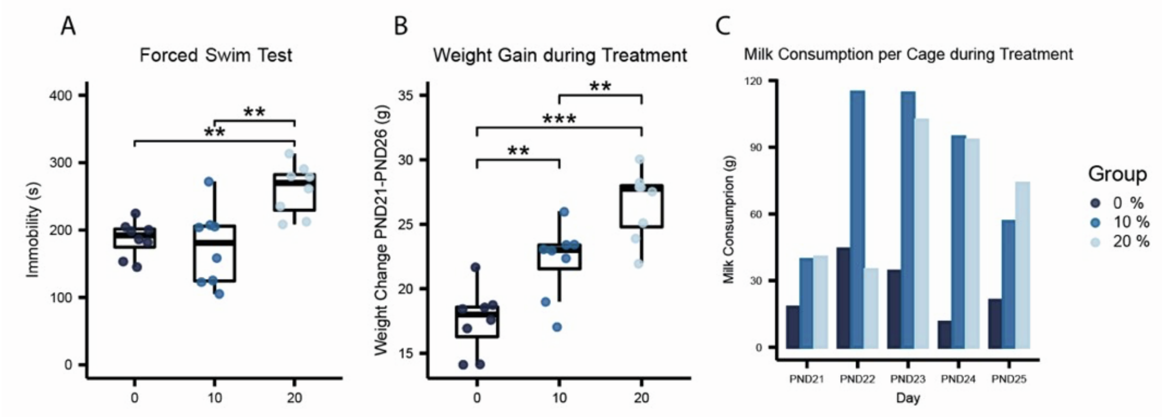

Figure S2. Concentration dependent effect of milk casein on milk consumption, body weight and stress-induced immobility behaviour, related to Figure 1
Casein-free milk formula (Special Diet services, Cambridge, UK) (0% casein) was spiked with 10% or 20% w/v casein (Sigma, Poole, UK) and provided to Male Wistar Albino rat pups upon weaning on PND21 until behavioural testing on PND25. A) Increased immobility time in 20% spiked milk group compared to both control and 10% spiked milk groups. No significant difference observed between control and 10% spiked milk group. B) Animals fed with 10% and 20% spiked milk gained significantly more weight compared to control. C) Animals appeared to consume per cage more spiked milk (both 10% w/w and 20% w/w) compared to casein-free milk. ANOVA followed by Tukey's HSD post-hoc test, significant p values are represented as ** $p < 0.01$, *** $p < 0.001$.

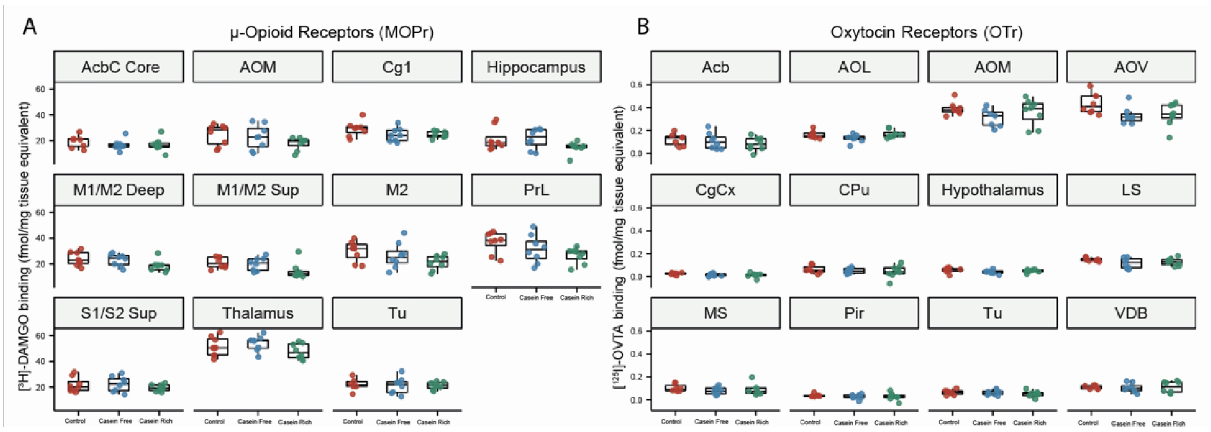

Figure S3. Brain autoradiography of mu-opioid and oxytocin receptors in Casein Study, related to Figure 1

Boxplots show first (lower) quartile, median and third (upper) quartile of A) mu-opioid receptors (MOPr) and B) oxytocin receptors (OTr). No significant differences were observed between groups (ANOVA followed by Tukey's HSD post-hoc test). AcbC, nucleus accumbens; AOM, anterior olfactory nucleusmedial; AOL, anterior olfactory nucleus-lateral; AOV, anterior olfactory nucleus-ventral; M1/M2 deep + supp, motor cortex deep or superficial respectively; M2, motor cortex; PrL, Prelimpic cortex; S1/S2 sup, somatosensory cortex superficial; Tu, olfactory tubercle; CgCx, cingulate cortex; CPu, caudateputamen; LS, lateral septum; MS, medial septum; Pir, piriform cortex; TU, Olfactory tubercle; VDB, vertical limb of the diagonal band Broca.

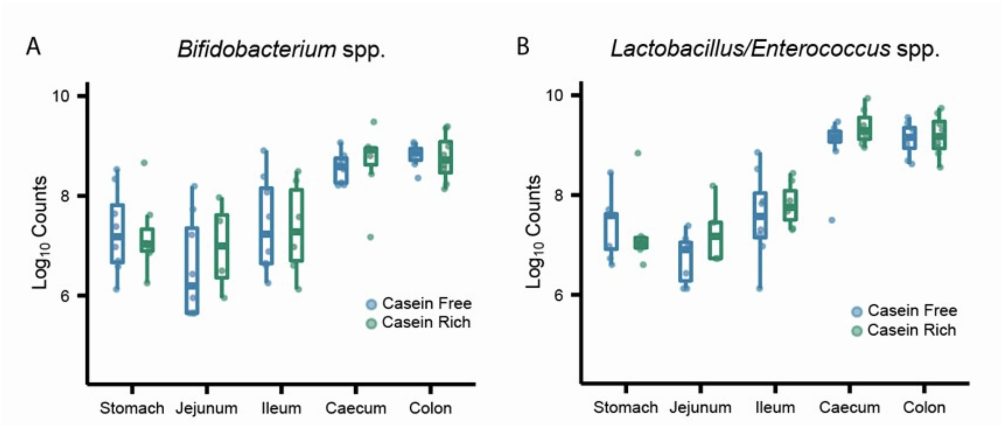

Figure S4. Fluorescenece *in-situ* hybridisation (FISH) analysis of gut contents collected from animals in the Casein Study, related to Figure 1

No significant differences were observed between treatments for *Bifidobacteria* and *Lactobacilli/Enterococci* populations in any of the regions analysed (Wilcoxon test followed by Benjamini-Hochberg correction).

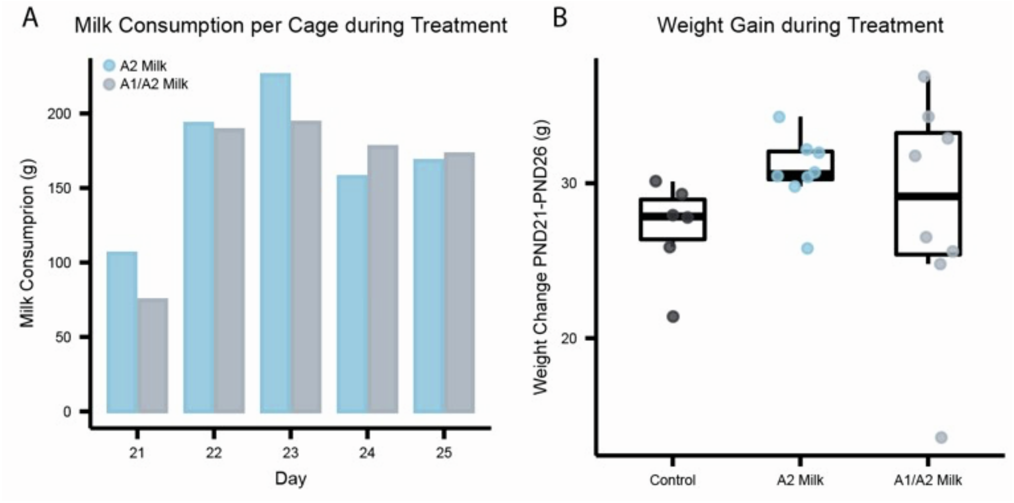

Figure S5. Milk consumption and weight gain during intervention in A1/A2 Study, related to Figure 3

A) No significant difference in milk consumption per cage was observed. B) No significant differences in weight gain between groups during treatment**.** ANOVA followed by Tukey's HSD post-hoc test.

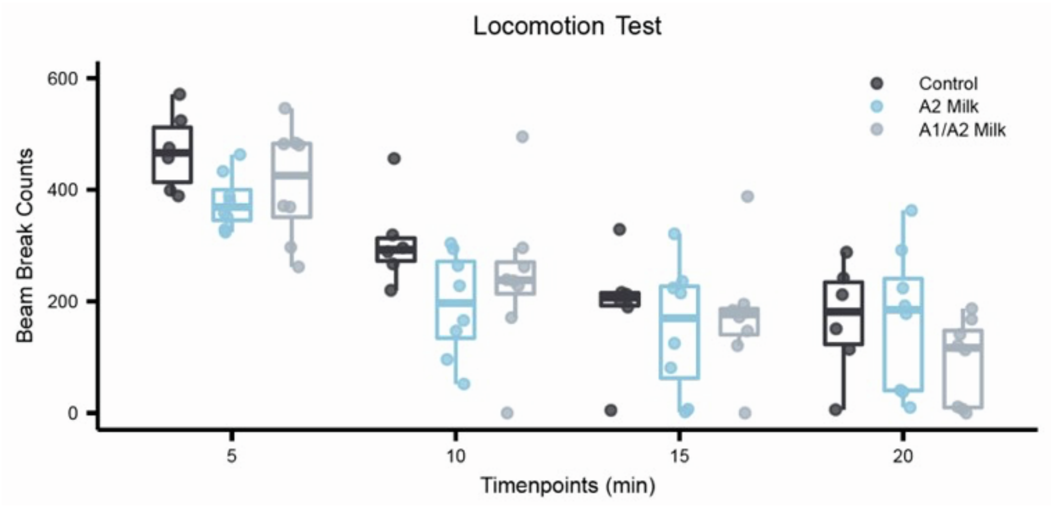

Figure S6. Locomotor activity test in A1/A2 Study, related to Figure 3

No significant differences were observed between the three groups at any of the timepoints studied. ANOVA followed by Tukey's HSD post-hoc test.

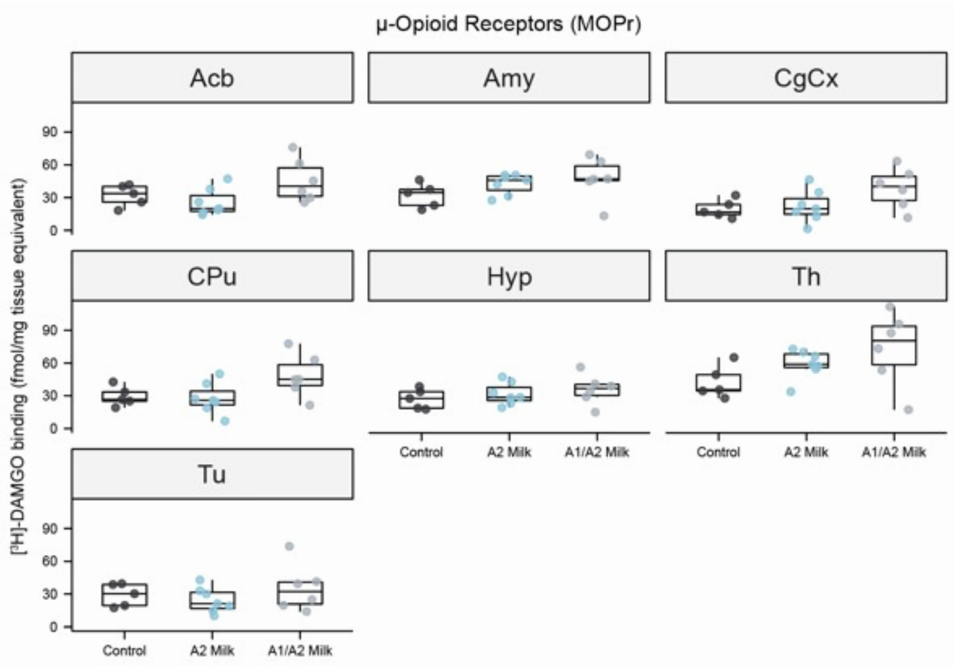

Figure S7. Brain autoradiography of mu-opioid receptors in A1/A2 Study, related to Figure 3
No significant differences were observed in any of the regions measured between the study groups (ANOVA followed by Tukey's HSD post-hoc test). Boxplots show first (lower) quartile, median and third (upper) quartile. AcbC, nucleus accumbens; Amy, amygdala; CgCx, cingulate cortex; CPu, caudate-putamen; Hyp, hypothalamus; Th, thalamus; TU, olfactory tubercle.

Casein Rich Study - Rodent Chow Formula (B&K Universal Ltd)

	Total Diet (w/w)	Units
Crude Oil	2.5	%
*Crude Protein *no casein*	18	%
Moisture, Crude Fiber, Carbohydrate, Nitrogen Free Extracts (NEF) and Other	74	%
Ash	5.5	%
Ingredients	Wheat meal, barley meal, soya meal, wheat feed, fish meal, fats and oils, minerals and trace elements, molasses, amino acids, vitamins, micro minerals	

A1/A2 Milk Study - Rodent Chow Formula (SDS, Kent UK)

	Total Diet (w/w)	Units
Crude Oil	2.71	%
*Crude Protein *no casein*	14.38	%
Moisture, Crude Fiber, Carbohydrate, Nitrogen Free Extracts (NEF) and Other	76.91	%
Ash	6	%
Ingredients	Wheat, barley, wheat feed, de-hulled extracted toasted, soya, soya protein concentrate, macro minerals, soya oil, whey powder, amino acids, vitamins, micro minerals	

Table S1. Rodent Chow formulation, related to Figure 1 and Figure 3
Table depicting the composition of the rodent chow diets provided during the Casein Rich Study and A1/A2 Milk Study.

Casein Rich Milk Formula

	Results Fresh Wight Milk (Liquid)	Units
Moisture	80.67	%
Fat	3.78	%
Protein	5.3 (4.24 Casein, 1.06 Whey)	%
Fibre	0.18	%
Ash Raw	1.24	%
NFE (Nitrogen Free Extract)	8.83	%
Ingredients	Spray dried skimmed milk, vegetable and fats mix, spray dried milk albumin, spray dried whey products, vitamins and minerals mix	

Casein Free Milk Formula

	Results Fresh Wight Milk (Liquid)	Units
Moisture	80.67	%
Fat	3.78	%
Protein	5.3 (4.24 Soy, 1.06 Whey)	%
Fibre	0.18	%
Ash Raw	1.24	%
NFE (Nitrogen Free Extract)	8.83	%
Ingredients	Whey powder, soy protein concentrate, maze oil, soya oil, vitamins and minerals mix	

A1/A2 Milk Formula

	Results Fresh Wight Milk (Liquid)	Units
Moisture	87.5	%
Fat	3.57	%
Protein	3.19 (2.55 Casein, 0.64 Whey)	%
Fibre	0	%
Ash Raw	0.7	%
NFE (Nitrogen Free Extract)	5.04	%

A2 Milk Formula

	Results Fresh Wight Milk (Liquid)	Units
Moisture	87.6	%
Fat	3.62	%
Protein	3.25 (2.6 Casein, 0.65 Whey)	%
Fibre	0	%
Ash Raw	0.7	%
NFE (Nitrogen Free Extract)	4.83	%

Table S2. Milk formula composition of the utilized milks, related to Figure 1 and Figure 3
Table depicting the composition of each of the milk diets used. Analysis of milk was carried out by Special Diet services, Cambridge UK. % casein and whey composition was calculated based on 80%/20% protein ratio in bovine milk
